# Supplementary material for: Transcriptomics provides a robust framework for the relationships of the major clades of cladobranch sea slugs (Mollusca, Gastropoda, Heterobranchia), but fails to resolve the position of the enigmatic genus Embletonia
Source: BMC Ecol Evol. 2021 Dec 28;21:226. doi: 10.1186/s12862-021-01944-0 (PMC8895541; doi:10.1186/s12862-021-01944-0)
Supplement: Supplementary file 1 — Additional file 1. Additional text. [file 12862_2021_1944_MOESM1_ESM.pdf]

Supplementary Text

to

Transcriptomics provides a robust framework for the relationships of the major clades of cladobranch sea slugs (Mollusca, Gastropoda, Heterobranchia), but fails to resolve the position of the enigmatic genus *Embletonia*

Dario Karmeinski, Karen Meusemann, Jessica A. Goodheart, Michael Schroedl, Alexander Martynov, Tatiana Korshunova, Heike Wägele, Alexander Donath*

*** Corresponding author**

Alexander Donath

Contact information:

a.donath@leibniz-zfmk.de

Table of Content

1. Taxon sampling..4

2. Sample preparation and sequencing...4

3. Quality checks and adapter trimming..5

4. *In silico* estimation of average insert sizes...5

5. Transcriptome assembly...5

6. Contaminant removal..9

7. Quality assessment of assemblies using BUSCO..9

8. Orthology assignment...10

9. Quality assessment after orthology assignment...11

10. Alignment...12

11. Outlier check..12

12. Removal of sequences from reference species and gap-only sites........................13

13. Filtering for ambiguous or randomly similar aligned sites, alignment masking, and concatenation into a supermatrix...13

14. Compilation, evaluation and optimization of data sets...14

15. Phylogenetic tree inference...16

16. Phylogenetic discordance..19

17. Investigation of the phylogenetic placement of *Embletonia pulchra*......................20

References...22

Note on taxon names: In several files and figures, some species names may differ from the names used here and in the main text due to identification updates and/or corrections. Valid names are found in the main text and Supplementary Table S1, Additional File 2. The following species are concerned:

**Name in analyses (outdated)**	**ID**	**Correct and valid name**	**Comment**
Aeolidiella alba	JG01	*Bulbaeolidia alba*	Taxonomic change
Antaeolidiella chromosoma	JG02	*Anteaeolidiella chromosoma*	Misspelling
Bathydoris clavigera	NC02	*Prodoris clavigera*	Taxonomic change
Calmella cavolinii	HW04	*Calmella cavolini*	Misspelling
Cuthona albocrusta	JG07	*Trinchesia albocrusta*	Taxonomic change
Cuthona caerulea	HW07	*Trinchesia morrowae*	Taxonomic change
Dendronotus orientalis	HW08	*Pseudobornella orientalis*	Taxonomic change
Flabellina babai	HW15	*Luisella babai*	Taxonomic change
Flabellina iodinea	JG16	*Flabellinopsis iodinea*	Taxonomic change
Hermissenda crassicornis	HW17	*Hermissenda emurai*	Misidentification
Janolus barbarensis	JG19	*Antiopella barbarensis*	Taxonomic change
Janolus cristatus	HW18	*Antiopella cristata*	Taxonomic change
Tritonia diomedea	NC07	*Tritonia tetraquetra*	Taxonomic change
Tritonia hamnerorum	NC08	*Tritonicula hamnerorum*	Taxonomic change
Tritonia manicata	HW21	*Duvaucelia manicata*	Taxonomic change
Unidentia angelvaldesi	JG32	*Unidentia* sp. 1	Misidentification

1. Taxon sampling

We collected samples of 19 species of Cladobranchia and two more distantly related species of heterobranch sea slugs from different locations in the Mediterranean Sea and the Sea of Japan between 2009 and April 2015 (see Supplementary Table S1, Additional File 2).

We complemented our data set with 40 previously published transcriptomes comprising 37 species of Cladobranchia as well as two dorids (*Prodoris clavigera, Doris kerguelenensis*) and one pleurobranchid (*Pleurobranchaea californica*) [1–4]. RNASeq data of the published samples were downloaded from the NCBI Sequence Read Archive (SRA) (see Supplementary Table S2, Additional File 2). Based on recently published findings [5], the transcriptome assigned to the species *Unidentia* angelvaldesi in [4] and [6] is indeed a new and undescribed species. We therefore use the name *Unidentia* sp. 1. Further names have been changed in the course of our analyses and are listed in detail above.

2. Sample preparation and sequencing

Newly collected specimens were preserved in RNAlater (Qiagen) and stored at -80 °C until further processing (Supplementary Table S1, Additional File 2). The specimens collected on Elba, Italy (sample IDs: HW05, HW07, HW09, HW11, HW12, HW13, HW16, HW18, HW19, HW21) were stored at -20 °C for approximately two weeks and then transferred to -80 °C until total RNA extraction. Two animals were preserved in IntactRNA from Evrogen (sample IDs: HW08, HW17). The extraction of total RNA was performed successively between December 2014 and spring 2015 using the Macherey-Nagel NucleoSpin RNA II kit. The preparation of the cDNA libraries and 150 bp paired-end sequencing were performed between January and June 2015 by StarSeq GmbH, Mainz, using the Illumina TruSeq Stranded RNA HT kit and the Illumina NextSeq 500 sequencing platform. We sequenced approximately 7.5 Gbases of raw data per sample. The unmodified raw reads of the newly sequenced transcriptomes are deposited under the BioProject PRJNA522208 in the NCBI

SRA (see Supplementary Table S2, Additional File 2 for accession numbers). The number of raw sequence reads and the average read length for each specimen are listed in Supplementary Table S3, Additional File 2.

3. Quality checks and adapter trimming

All raw sequence reads were quality-checked prior to and after adapter trimming using FastQC v. 0.11.5 [7]. Adapter trimming and quality filtering were performed with Trimmomatic v. 0.36 [8] using a custom-made adapter file containing all Illumina adapters. Trimmomatic was run with the following parameters: `adapters.fa:2:30:9:1:true MAXINFO:80:0.5 MINLEN:80`.

The number of the raw reads and average read lengths for the trimmed data of each sample are listed in Supplementary Table S3, Additional File 2. The adapter file with the Illumina adapters used for library preparation can be found in Additional File 4.

4. *In silico* estimation of average insert sizes

Some *de novo* transcriptome assemblers, such as SOAPdenovo-Trans [9], require the average insert size of the raw reads of an input library. The average insert sizes were estimated with the software BBMerge as implemented in the package BBTools version 37.28 [10, 11], rounded to the closest integer and provided to SOAPdenovo-Trans as input (Supplementary Table S3, Additional File 2).

5. Transcriptome assembly

All 61 transcriptomes from the newly sequenced and previously published samples were *de novo* assembled using six commonly used assembly programs: BinPacker version 1.1 [12],

IDBA-Tran v. 1.1.1 [13], Shannon v. 0.0.2 [14], SOAPdenovo-Trans v. 1.04 [9], Trans-ABySS v. 1.5.5 [15], and Trinity v. 2.4.0 [16]. All assemblers were run with default settings with the paired-end reads that survived the trimming process. Additionally, we provided single-end reads that had survived trimming to those assemblers capable of processing them (i.e. IDBA-Tran, SOAPdenovo-Trans, and Trans-ABySS).

The assembly process of the 61 raw data sets resulted in a total of 366 (six assemblies per sample) transcriptome assemblies. The number of assembled contigs and total size of each assembly are provided in Supplementary Table S4, Additional File 2.

BinPacker

BinPacker was run with the following parameters:

```
BinPacker -s fq -p pair -m FR -l R1_pd.fastq -r R2_pd.fastq -u both.fastq
```

Although it is possible to pass both paired-end and single-end reads to BinPacker in the same run, the generated log files indicated that only paired-end reads had been used in the *de novo* assembly process, which we assume to be an unsolved issue in BinPacker.

IDBA-Tran

IDBA-Tran was run with the following parameters:

```
idba_tran -l all.fa -o [OUT] --num_threads [NUM_THREADS]
```

IDBA-Tran requires input reads to be in FASTA format. Therefore, the included conversion software `fq2fa` was run with the following parameters for paired-end reads:

```
fq2fa --merge --filter R1_pd.fastq R2_pd.fastq R1_R2.fa
```

And for single-end reads:

```
fq2fa SE.fastq SE.fa
```

Subsequently, all reads were combined into a single file.

Shannon

Shannon was run with the following parameters:

```
shannon.py -o [TAXON] --left R1_pd.fastq --right R2_pd.fastq -p [NUM_THREADS]
```

Because Shannon can only process paired-end reads, no single reads were used in the assembly process.

SOAPdenovo-Trans

SOAPdenovo-Trans was run with the following parameters:

```
SOAPdenovo-Trans-127mer all -s config.txt -o soap -p [NUM_THREADS]
```

SOAPdenovo-Trans requires a config file (example provided below). For each sample, the value [AVG_INS] is replaced with the corresponding average insert size value estimated by BBMerge (see Supplementary Table S3, Additional File 2).

```
max_rd_len=151
```

```
[LIB]
rd_len_cutof=151
avg_ins=[AVG_INS]
reverse_seq=0
asm_flags=3
map_len=32
q1=PATH/R1_pd.fastq
q2=PATH/R2_pd.fastq
q=PATH/both.fastq
```

Trans-ABySS

Trans-ABySS was run with the following parameters:

```
transabyss --pe R1_pd_Trans-AByss.fastq R2_pd_Trans-AByss.fastq --se both.fastq --outdir [OUT] --mpi [NUM_THREADS] --threads [NUM_THREADS]
```

Trans-ABySS requires sequence headers to have an additional “/1” or “/2” at the end to identify forward and reverse reads. Therefore, all sequence headers were adjusted accordingly prior to the assembly process using a custom Perl script.

Trinity

Trinity was run with the following parameters:

```
Trinity --seqType fq --left R1.fastq --right R2.fastq --CPU [NUM_THREADS] --SS_lib_type FR --max_memory 100G --trimmomatic --quality_trimming_params ILLUMINACLIP:adapters.fa:2:30:9:1:true MAXINFO:80:0.5 MINLEN:80
```

Note that untrimmed reads were used as input and trimming was performed by Trinity employing Trimmomatic within the Trinity package using the same parameters as listed in section 3.

6. Contaminant removal

Following the identification of the best transcriptome assembly per species (see below), possible foreign contaminants (non-target sequences, adapter/linker sequences) within the assemblies of the newly sequenced samples were identified upon submission to NCBI Transcriptome Shotgun Assembly (TSA) database. Terminal strong or moderate contaminating sequence regions were clipped from the respective sequence. In cases of internal matches, the potential sequence contamination was removed and the transcript split into two separate sequences. All sequences of at least 200 bp were submitted to the NCBI TSA database. Supplementary Table S7, Additional File 2 lists details of the contaminant removal for all newly sequenced species. Note that *Pseudobornella orientalis* was later excluded from further analyses (and also removed from TSA) due to an exceptionally low sequence quality (see section 8 and Supplementary Table S7, Additional File 2). The five alternative assemblies of all other species that have been newly sequenced for this study are available in Additional File 5.

7. Quality assessment of assemblies using BUSCO

We checked the quality of our transcriptomes by using BUSCO version 3.0.0 [17] with the reference data set "metazoa".

The following settings used were:

```
run_BUSCO.py -f -i assembly.fasta -c 12 -l metazoa_odb9 -m tran
```

The highest BUSCO value was 943 complete BUSCO genes out of 978 (fragmented: 27, missing: 8) in *Caloria elegans* assembled with BinPacker and the lowest one was 158 complete BUSCO genes (fragmented: 123 missing: 697) in *Doris kerguelenensis*, also assembled with BinPacker. The median was 731 complete BUSCO genes. BUSCO values for all assemblies are listed in Supplementary Table S5, Additional File 2.

To further judge the quality of our assemblies, we additionally performed a second check after orthology assignment, see section 9.

8. Orthology assignment

In order to set up a reference database for the software Orthograph [18], which we used to infer orthology for our transcripts, we selected a set of clusters of orthologous sequences of 1,997 single-copy protein-coding genes (ortholog groups; OGs) from OrthoDB version 9 [19]. Setting the hierarchical level to “Lophotrochozoa”, we considered the official gene sets (OGS) of all three reference species with well-sequenced and annotated genomes present in OrthoDB: *Biomphalaria glabrata*, Official Gene Set (OGS) version 1.2 vectorbase [20], *Crassostrea gigas*, OGS version Sep-2012 (ENA genebuild) [21], and *Lottia gigantea*, OGS version Jan-2013 (JGI genebuild) [22]. From this ortholog set, we excluded five genes because of inconsistent sequence headers. Thus, a total of 1,992 OGs were used to assign transcripts of each sample (Additional File 6).

Orthology assignment for transcripts included in the assembled transcriptomes was then performed using Orthograph version 0.6.2 [18]. An example Orthograph configuration file with the settings that were used is available in the supplementary material (Additional File 6). In short, we used default settings allowing the reciprocal search of candidate orthologous transcripts against one of all official gene sets of the reference species. Furthermore, we allowed concatenation of transcripts in case they matched one OG, but did not overlap. We

were able to successfully assign at least one transcript for 1,989 out of 1,992 OGs, for three OGs no transcript was assigned; these three OGs were excluded from further processing.

9. Quality assessment after orthology assignment

Since the BUSCO set "metazoa" is not very specific with respect to Cladobranchia, and since there is no specific sea-slug data set available yet, we additionally established a second check for our study: The Python script *Orthograph_Quality_Checker.py*, which analyzes the set of amino acid sequences that have been successfully assigned to an OG by Orthograph for a given assignment run, i.e. all transcript sequences of an assembled transcriptome. For each output of an Orthograph run (one run per sample and per assembler), it calculates three characteristics: i) the number of sequences that were assigned to OGs, ii) the cumulative length of these sequences, and iii) the overlap (in %) between the cumulative length of transcripts assigned to OGs and the average lengths of the corresponding reference sequences (see Supplementary Table S6, Additional File 2). We used the second value as a criterion to select the best assembly per sample, i.e. the assembly that provided the most complete sequences from the OGs. The script, an example config, and an example results file are available on github (https://github.com/alexdonath/Embletonia). Note that there was no general tendency with respect to a "best" or "worst" performing assembler.

To reduce the amount of missing data per species, we only kept taxa that matched at least 60% of all included OGs of the ortholog set. Based on this criterion, we excluded *Pseudobornella orientalis* (HW08, 53%), *Dermatobranchus* sp. (JG09, 46%), and *Tritoniopsis frydis* (JG31, 51%) from further processing. Next, we only kept OGs, for which at least 50% of the investigated species had a positive hit. This resulted in 1,767 OGs for further analyses.

Subsequently, sequence headers were converted with the script `orthograph2hamstrad.pl` provided with the Orthograph package. Orthograph results were summarized on amino acid level with the script `summarize_orthograph_results.pl` (options `-t`, `-m`, and `-u`) to create one multiple sequence file per OG containing transcripts of all transcriptome species and the three reference species.

10. Alignment

Translated sequences were obtained from the Orthograph output and were used to generate multiple sequence alignments on amino acid level of each OG using DIALIGN-TX Version 1.0.2 [23] with the following parameters:

```
dialign-tx [conf-directory] [FILE IN] [FILE OUT]
```

All alignments are available in Additional File 7.

11. Outlier check

Multiple sequence alignments were checked for outlier sequences (i.e. putatively misaligned or misassigned amino acid sequences) following the method described in [24]. We chose, however, to reimplement the method with some notable modifications. Our reimplementation now allows for arbitrary sets of reference taxa. Furthermore, we based the outlier identification on a formal definition of the interquartile range (IQR). The script is available from github [25] and has been run with the following command:

```
checker_complete.2.pl -a [ALIGNMENT] -s subjects.txt -k
```

Given the evolutionary distance of the three reference species, which we expect to be higher than the genetic distance within Cladobranchia, we considered their sequence dissimilarity as a cutoff for the selection of outlier sequences.

Identified outlier sequences were subsequently removed from all amino acid alignments (custom script available from: https://github.com/alexdonath/Embletonia). Altogether, we discarded 897 sequences from 112 multiple sequence alignments. Outliers were found in sequences from all remaining 58 species with the highest number being 30 outliers in *Limenandra confusa* and the lowest being eight outliers in *Doris kerguelenensis*. The number of outliers for every species is available in Supplementary Table S8, Additional File 2.

12. Removal of sequences from reference species and gap-only sites

After the removal of identified outlier sequences, we further removed all sequences from the reference species *Biomphalaria glabrata*, *Crassostrea gigas*, and *Lottia gigantea* from the alignments using the custom Python script `remove_reference_sequences.py` (available from: https://github.com/alexdonath/Embletonia). Subsequently, gap-only sites were removed from each multiple sequence alignment.

13. Filtering for ambiguous or randomly similar aligned sites, alignment masking, and concatenation into a supermatrix

The amino acid multiple sequence alignments were examined with the program Aliscore version 2.0 [26, 27] with default settings in order to identify ambiguous or randomly similar aligned sites. All positions flagged by Aliscore per gene were discarded using the Perl script AliCUT version 2.31 [28]. For 248 genes masking was not necessary, so 1,519 gene

alignments were masked. The maximal proportion that was discarded from an MSA was 93.3%.

The resulting masked amino acid multiple sequence alignments were concatenated into a single supermatrix using FASconCAT-G version 1.04 [29] and a corresponding partition file based on the OGs was created. This supermatrix, which we hereinafter call "original unreduced data set", comprised 58 species, spanned a length of 771,739 positions (~ 71% of the unmasked superalignment), and contained 1,767 gene partitions.

14. Compilation, evaluation and optimization of data sets

Based on our original unreduced data set, we compiled another supermatrix, hereinafter called "original reduced data set", with the aid of the Perl script `fasta2hypo` (see Supplement of [24]) and kept only those gene partitions with sequence data for all 58 species. Thus, we ensured 100% partition coverage for each included species. The original reduced data set comprised 143,859 aligned amino acid positions and 364 gene partitions.

We analyzed both data sets with the software tool MARE version 1.2-rc [30] in order to assess the potential information content (IC) of each partition, the overall information content, and the coverage in terms of gene partitions. Subsequently, AliStat v. 1.6 [31] was used to calculate alignment diagnostics of the supermatrices. Finally, the software SymTest v. 2.0.47 [32–34] was used to analyze among-lineage compositional heterogeneity of the supermatrices in order to detect possible model violations of the stationary, (time-)reversible and homogeneous (SRH) conditions using the implemented Bowker's test of matched paired symmetry [35].

Considering the original reduced data set, SymTest revealed a much stronger violation of SRH conditions for the two species *Doris kerguelenensis* and *Calmella cavolini* compared to all other taxa included. Thus, we excluded both *Doris kerguelenensis* and *Calmella cavolini*

and therefore removed all sequences of both species from each of the original 1,767 multiple sequence alignments. Subsequently, all remaining gap-only sites were removed as well. All final MSAs are available in Additional File 8.

We repeated the concatenation into supermatrices and generated again i) a data set in which all MSAs were combined in a supermatrix, termed "unreduced data set" and ii) a reduced data set based on the same criterion as described above, termed "strict data set". Further, we compiled iii) a data set that contained only those gene partitions for which at least one representative of each of the defined groups was present, termed "intermediate data set". Group definitions and included species for this data set are given in Supplementary Table S9, Additional File 2. Finally, we increased the overall information content (IC) of the strict data set using MARE with default settings. By discarding less informative genes as identified by MARE this resulted in iv) a strict selected optimal subset (SOS). All four data sets including the respective gene partition information are available in Additional File 9.

Note that we consider the strict data set as our main data set for two reasons: first, the amount of missing data is minimized and the overlap of all taxa is maximized following the rationale of Dell'Ampio and colleagues [36] (Supplementary Table S11, Additional File 2) and secondly, the amount of data violating SRH model assumptions was massively reduced (compare Supplementary Figures S1a and b, Additional File 3; Supplementary Figures S2a and b, Additional File 3).

We then re-calculated the overall information content with MARE, AliStat supermatrix diagnostics, and SymTest pairwise sequences scores generating heatmaps for the four final data sets. These comprised 56 species; the unreduced data set spanned 771,707 amino acid positions and included 1,767 gene partitions; the intermediate data set spanned 271,732 amino acid positions and included 667 gene partitions; the strict data set spanned

170,140 amino acid positions and included 446 gene partitions; and the strict SOS data set spanned 126,094 amino acid positions and included 335 gene partitions. Detailed diagnostics of the three final data sets are provided in Supplementary Tables S10 and S11, Additional File 2, heatmaps generated with MARE, AliStat, and SymTest are displayed in Supplementary Figures S3-S8, Additional File 3.

15. Phylogenetic tree inference

For all four final data sets (unreduced, intermediate, strict, and strict SOS), phylogenetic trees were calculated under the maximum likelihood optimality criterion using the program IQ-TREE version 1.6.12 [37]. The best fitting amino acid models were identified using ModelFinder [38], which is implemented in IQ-TREE. To this end, ModelFinder was run with an edge-linked partitioned approach [39]. Models were selected from all included nuclear models with equal rates (E), gamma rates (G), gamma and invariant sites (G+I), and free rates (R) with 2 - 15 categories as well as the two free-rate models LG4X and LG4M, which by default only have four categories [40]. The model for each partition was chosen based on the corrected Akaike Information Criterion (AICc) [41]. Details about the selected substitution models are provided in Additional File 9.

Additionally, we ran a mixture model analysis for the unpartitioned strict data set using IQ-TREE version 1.6.12. ModelFinder was used to select the best mixture model. This was done by first performing an extended model selection on the unpartitioned strict data set to identify the best fitting amino acid exchange rate matrix. Models were selected from all included nuclear models with equal rates (E), gamma rates (G), gamma and invariant sites (G+I), and free rates (R) with 2 - 8 categories as well as the two free-rate models LG4X and LG4M. In a next step, the three best-performing matrices were tested against and in combination with all protein mixture models (as implemented in IQ-TREE version 1.6.12), 10, 20, 30, and 60 classes of amino acid profiles (C10, C20, C30, and C60), and using empirical

base frequencies (F). The final round of selecting the best mixture model tested the best-performing amino acid exchange rate matrix in combination with 20, 30, and 60 classes of amino acid profiles (C20, C30, and C60) and using empirical (F) as well as optimized based frequencies (FO) and 4 - 6 rate categories (R4 - R6). In each step, the best model was chosen based on the AICc.

For each data set, a total of 20 tree searches were performed, out of which ten were run using a parsimonious start tree and ten using a random start tree.

The best maximum likelihood tree was selected according to the best log-likelihood value. Statistical support was inferred from non-parametric bootstrap replicates in batches of 100 replicates. For all data sets 100 bootstrap replicates were calculated. Since 100 replicates were not sufficient to achieve bootstrap convergence for the intermediate and strict data set, for both data sets 300 bootstrap replicates were calculated (see below). Convergence of bootstrap replicates was ensured with RAxML version 8.2.11 [42] using the following options: `-I autoMRE -B 0.03 -m GTRGAMMA` and 10,000 permutations. Testing for bootstrap convergence was conducted ten times independently with random start seeds. For the unreduced and the unpartitioned strict data set, bootstrap convergence was always achieved after 50 replicates, whereas bootstraps of the intermediate data set and the partitioned strict data set required 150 replicates each to reach convergence. For the strict SOS data set, bootstrap convergence was achieved four times after 100 BS replicates and six times after 50 replicates.

Statistical bootstrap support was mapped on the ML best tree of each analysis, i.e. unreduced, intermediate, partitioned strict, unpartitioned strict, and strict SOS, respectively (Fig. 1 and Supplementary Figures S10, S12, S14, and S16, Additional File 3).

In addition to bootstrap support, we calculated support values based on the SH-like approximate likelihood ratio test [43] with 10,000 replicates and based on the approximate Bayes test [44] using default settings and the best inferred ML tree for each data set (unreduced, intermediate, partitioned strict, unpartitioned strict, and strict SOS) as input (Supplementary Figures S9, S11, S13, S15, and S17, Additional File 3).

All trees were rooted in Dendroscope v. 3.7.2 [45] by using the clade containing *Berthella plumula* and *Pleurobranchaea californica* as an outgroup. Furthermore, the tree was graphically edited with Graphic for Mac v. 3.1. FigTree v. 1.4.4 [46] was used to collapse nodes in Fig. 2.

The number of unique best ML tree topologies for each of the five analyses was assessed using Unique Tree v. 1.9 kindly provided by Thomas Wong. For each data set, except the unpartitioned strict data set analysed using a mixture model approach, we only found one unique tree topology. For the unpartitioned strict data set we found three different tree topologies, differing in the position of *Caloria elegans* within Facelinidae:

Topology 1 (found 13 times): (*Learchis evelinae*, (*Caloria elegans*, (*Phidiana lynceus*, (*Austraeolis stearnsi*, *Palisa papillata*))))

Topology 2 (found four times): (*Caloria elegans*, (*Learchis evelinae*, (*Phidiana lynceus*, (*Austraeolis stearnsi*, *Palisa papillata*))))

Topology 3 (found three times): (*Learchis evelinae*, (*Phidiana lynceus*, (*Caloria elegans*, (*Austraeolis stearnsi*, *Palisa papillata*))))

The five final data sets (unreduced, intermediate, partitioned strict, unpartitioned strict, and strict SOS) were tested for the presence of rogue taxa using RogueNaRok v. 1.0 [47] with default settings, to which the best ML tree inferred from each data set was provided as an input. No rogue taxa were identified in any of the data sets.

16. Phylogenetic discordance

To further analyse phylogenetic discordance, we applied the Quartet Sampling (QS) method [48]. The QS method aims to identify a lack of branch support due to low phylogenetic information, discordance because of lineage sorting or introgression, and misplaced or erroneous taxa (rogue taxa) by repeatedly and randomly sampling one taxon from each of the four subsets at a focal branch. For each quartet sampled, the likelihood is evaluated for all three possible topologies given the sequence data for the randomly selected quartet, and the frequencies, with which either the concordant or one of the two discordant topologies shows the best likelihood, are counted. These counts are then used to calculate three branch scores to quantify the relative support among the three possible topologies of four taxa (quartet concordance, QC), the disparity between the sampled proportions of the two discordant topologies (quartet differential, QD), and the proportion of replicates where the likelihood value of the best-likelihood quartet tree exceeds the second-best likelihood score by a given threshold (quartet informativeness, QI).

The QS analysis was conducted on the tree inferred from the (partitioned) strict data set with a maximum of 100 quartet replicates. The likelihood of the three possible topologies for each quartet sample was evaluated using RAxML v. 8.2.12 [42], using the same models used for ML tree inference with IQ-TREE (see above).

The detailed results of the QS analysis are given in the Supplementary Material (Supplementary Table S12, Additional File 2 and Additional File 10).

17. Investigation of the phylogenetic placement of *Embletonia pulchra*

Since the position of *Embletonia pulchra* was not stable between the ML trees calculated from the five data sets, we performed approximately unbiased (AU) tests [49] on the intermediate, partitioned strict, the unpartitioned strict, and the strict SOS data set as implemented in IQ-TREE version 1.6.12. Note that the position of *E. pulchra* was identical in the best ML trees from the unreduced and the partitioned strict data set as well as in the best ML trees from the intermediate, the unpartitioned strict, and the strict SOS data set. AU tests were performed with 100,000 RELL replicates [50] using the best tree inferred for each data set to optimize model parameters (option `-te`). Therefore, the best ML trees were modified manually by changing the position of *Embletonia* with the aid of Mesquite v. 3.61 [51].

In particular, we tested (see Fig. 2):

i) *Embletonia* as sister to Proctonotoidea (suggested by the best ML trees from the partitioned strict and unreduced data set)

ii) *Embletonia* as sister to Aeolidida (suggested by the best ML tree from the intermediate, the unpartitioned strict, and the strict SOS data set)

iii) *Embletonia* as sister to the clade Proctonotoidea + Aeolidida

Note that we also tested these three positions using the Four-cluster Likelihood Mapping approach (FcLM, see below) on the strict data set.

In addition, we tested four more positions due to putative relationships suggested by various literature applying the AU test:

iv) *Embletonia* is sister to Dendronotoidea

v) *Embletonia* is sister to Arminidae

vi) *Embletonia* is sister to Fionoidea

vii) *Embletonia* is sister to Unidentiidae (and this clade is sister to remaining Fionoidea)

All tree topologies are found in Additional File 11.

The AU test on the partitioned strict data set did not reject a placement of *Embletonia* as sister to Aeolidida (as inferred in analysis of the intermediate, the unpartitioned strict, and the strict SOS data set), nor did the AU test on the intermediate, unpartitioned strict, and strict SOS data set reject the placement of *Embletonia* as sister to Proctonotoidea (as inferred in the analysis of the unreduced and strict data set; $p > 0.05$, see Supplementary Table S15, Additional File 2). All other alternative positions of *Embletonia* (tested only with the strict data set) were significantly rejected.

Additionally, Four-cluster Likelihood Mapping (FcLM) [52] was performed on the strict data set, which we consider as our main data set, because it has full partition coverage for each included species and the least amount of missing data. We assessed the strict data set for conflicting signal in order to address the placement of *Embletonia* as sister to Proctonotoidea and to explore this data set for possible confounding signal that might originate from model violation of SRH conditions and/or non-randomly distributed data (for a rationale, see [24, 53, 54]). For this purpose, four groups were defined as follows:

Group 1 = *Embletonia pulchra* (1 species)

Group 2 = Proctonotoidea (*Dirona* + *Antiopella*, 3 species)

Group 3 = Aeolidida (33 species)

Group 4 = Tritoniidae, Arminidae, Dendronotoidea + outgroup taxa (19 species)

Groups and included species are provided in Supplementary Table S13, Additional File 2.

In a second FcLM analysis, we slightly modified Group 4 by excluding the (non-cladobranch) outgroup taxa *Pleurobranchaea*, *Berthella*, *Polycera*, and *Prodoris*.

We performed FcLM analysing all possible quartets on the original data set (i.e. two analyses with Group 4 being modified) with partition boundaries and selected models as used for ML tree inference with IQ-TREE v. 1.6.9. We furthermore applied three permutations to identify putative confounding signal [24, 54]. All data for the FcLM analyses are available in Additional File 12. Detailed results are provided in Supplementary Table S14, Additional File 2.

Irrespective of whether or not the outgroup taxa were excluded from Group 4, the results remain ambiguous: The majority of quartets suggest a placement of *Embletonia* as sister to a clade Proctonotoidea + Aeolidia. Only less than 20% of the quartets supported *Embletonia* as sister to Proctonotoidea, as inferred in our best ML tree from the strict data set. However, *Embletonia* as sister to Proctonotoidea can be fully explained by confounding signal, as indicated by the permutation approach. This implies that confounding signal most likely outperformed phylogenetic signal and affected ML tree inference. However, the topology suggested by the majority of the quartets, i.e. *Embletonia* as sister to Proctonotoidea + Aeolidia, was rejected by the AU test. Therefore, we cannot draw conclusions and consider the placement of *Embletonia* as unresolved, which is also reflected in the negligible statistical support in the inferred ML trees from the strict and intermediate data sets.

References

1. Zapata F, Wilson NG, Howison M, Andrade SCS, Jörger KM, Schrödl M, et al. Phylogenomic analyses of deep gastropod relationships reject Orthogastropoda. Proc R Soc Lond B Biol Sci. 2014;281:20141739.

2. Goodheart JA, Bazinet AL, Collins AG, Cummings MP. Relationships within Cladobranchia (Gastropoda: Nudibranchia) based on RNA-Seq data: an initial investigation. R Soc Open Sci. 2015;2:150196.

3. Senatore A, Edirisinghe N, Katz PS. Deep mRNA sequencing of the *Tritonia diomedea* brain transcriptome provides access to gene homologues for neuronal excitability, synaptic transmission and peptidergic signalling. PLOS ONE. 2015;10:e0118321.

4. Goodheart JA, Bazinet AL, Valdés Á, Collins AG, Cummings MP. Prey preference follows phylogeny: evolutionary dietary patterns within the marine gastropod group Cladobranchia (Gastropoda: Heterobranchia: Nudibranchia). BMC Evol Biol. 2017;17:221.

5. Korshunova T, Mehrotra R, Arnold S, Lundin K, Picton B, Martynov A. The formerly enigmatic Unidentiidae in the limelight again: a new species of the genus *Unidentia* from Thailand (Gastropoda: Nudibranchia). Zootaxa. 2019;4551:556.

6. Goodheart JA, Bleidißel S, Schillo D, Strong EE, Ayres DL, Preisfeld A, et al. Comparative morphology and evolution of the cnidosac in Cladobranchia (Gastropoda: Heterobranchia: Nudibranchia). Front Zool. 2018;15:43.

7. Andrews S. FastQC: a quality control tool for high throughput sequence data. 2010. http://www.bioinformatics.babraham.ac.uk/projects/fastqc.

8. Bolger AM, Lohse M, Usadel B. Trimmomatic: a flexible trimmer for Illumina sequence data. Bioinformatics. 2014;30:2114–20.

9. Xie Y, Wu G, Tang J, Luo R, Patterson J, Liu S, et al. SOAPdenovo-Trans: de novo transcriptome assembly with short RNA-Seq reads. Bioinformatics. 2014;30:1660–6.

10. Bushnell B, Rood J, Singer E. BBMerge – Accurate paired shotgun read merging via overlap. PLOS ONE. 2017;12:e0185056.

11. Bushnell B. BBMap. sourceforge.net/projects/bbmap/.

12. Liu J, Li G, Chang Z, Yu T, Liu B, McMullen R, et al. BinPacker: Packing-based *de novo* transcriptome assembly from RNA-seq sata. PLOS Comput Biol. 2016;12:e1004772.

13. Peng Y, Leung HCM, Yiu S-M, Lv M-J, Zhu X-G, Chin FYL. IDBA-tran: a more robust de

novo de Bruijn graph assembler for transcriptomes with uneven expression levels. Bioinformatics. 2013;29:i326–34.

14. Kannan S, Hui J, Mazooji K, Pachter L, Tse D. Shannon: An information-optimal de novo RNA-Seq assembler. bioRxiv. 2016;:039230.

15. Robertson G, Schein J, Chiu R, Corbett R, Field M, Jackman SD, et al. De novo assembly and analysis of RNA-seq data. Nat Methods. 2010;7:909–12.

16. Grabherr MG, Haas BJ, Yassour M, Levin JZ, Thompson DA, Amit I, et al. Full-length transcriptome assembly from RNA-Seq data without a reference genome. Nat Biotechnol. 2011;29:644–52.

17. Simão FA, Waterhouse RM, Ioannidis P, Kriventseva EV, Zdobnov EM. BUSCO: assessing genome assembly and annotation completeness with single-copy orthologs. Bioinformatics. 2015;31:3210–2.

18. Petersen M, Meusemann K, Donath A, Dowling D, Liu S, Peters RS, et al. Orthograph: a versatile tool for mapping coding nucleotide sequences to clusters of orthologous genes. BMC Bioinformatics. 2017;18:111.

19. Kriventseva EV, Tegenfeldt F, Petty TJ, Waterhouse RM, Simão FA, Pozdnyakov IA, et al. OrthoDB v8: update of the hierarchical catalog of orthologs and the underlying free software. Nucleic Acids Res. 2015;43:D250–6.

20. DeJong RJ, Emery AM, Adema CM. The mitochondrial genome of *Biomphalaria glabrata* (Gastropoda: Basommatophora), intermediate host of *Schistosoma mansoni*. J Parasitol. 2004;90:991–7.

21. Zhang G, Fang X, Guo X, Li L, Luo R, Xu F, et al. The oyster genome reveals stress adaptation and complexity of shell formation. Nature. 2012;490:49–54.

22. Simakov O, Marletaz F, Cho S-J, Edsinger-Gonzales E, Havlak P, Hellsten U, et al. Insights into bilaterian evolution from three spiralian genomes. Nature. 2013;493:526–31.

23. Subramanian AR, Kaufmann M, Morgenstern B. DIALIGN-TX: greedy and progressive approaches for segment-based multiple sequence alignment. Algorithms Mol Biol. 2008;3:6.

24. Misof B, Liu S, Meusemann K, Peters RS, Donath A, Mayer C, et al. Phylogenomics resolves the timing and pattern of insect evolution. Science. 2014;346:763–7.
25. Donath A. Checker complete v2. 2019. https://github.com/alexdonath/checker_complete-v2.
26. Misof B, Misof K. A Monte Carlo approach successfully identifies randomness in multiple sequence alignments: a more objective means of data exclusion. Syst Biol. 2009;58:21–34.
27. Kück P, Meusemann K, Dambach J, Thormann B, von Reumont BM, Wägele JW, et al. Parametric and non-parametric masking of randomness in sequence alignments can be improved and leads to better resolved trees. Front Zool. 2010;7:10.
28. Kück P. AliCUT. 2019. https://github.com/PatrickKueck/AliCUT.
29. Kück P, Longo GC. FASconCAT-G: extensive functions for multiple sequence alignment preparations concerning phylogenetic studies. Front Zool. 2014;11:81.
30. Misof B, Meyer B, von Reumont BM, Kück P, Misof K, Meusemann K. Selecting informative subsets of sparse supermatrices increases the chance to find correct trees. BMC Bioinformatics. 2013;14:348.
31. Wong TKF, Kalyaanamoorthy S, Meusemann K, Yeates DK, Misof B, Jermiin LS. A minimum reporting standard for multiple sequence alignments. NAR Genomics Bioinforma. 2020;2. doi:10.1093/nargab/lqaa024.
32. Ho SYW, Jermiin LS. Tracing the decay of the historical signal in biological sequence data. Syst Biol. 2004;53:623–37.
33. Ababneh F, Jermiin LS, Ma C, Robinson J. Matched-pairs tests of homogeneity with applications to homologous nucleotide sequences. Bioinformatics. 2006;22:1225–31.
34. Jermiin LS, Ott M. SymTest. C++. 2017. https://github.com/ottmi/symtest. Accessed 28 May 2020.
35. Bowker AH. A test for symmetry in contingency tables. J Am Stat Assoc. 1948;43:572–4.
36. Dell'Ampio E, Meusemann K, Szucsich NU, Peters RS, Meyer B, Borner J, et al. Decisive data sets in phylogenomics: lessons from studies on the phylogenetic relationships

of primarily wingless insects. Mol Biol Evol. 2014;31:239–49.

37. Nguyen L-T, Schmidt HA, Haeseler A von, Minh BQ. IQ-TREE: A fast and effective stochastic algorithm for estimating maximum-likelihood phylogenies. Mol Biol Evol. 2015;32:268–74.

38. Kalyaanamoorthy S, Minh BQ, Wong TKF, von Haeseler A, Jermiin LS. ModelFinder: fast model selection for accurate phylogenetic estimates. Nat Methods. 2017;14:587–9.

39. Chernomor O, Haeseler A von, Minh BQ. Terrace aware data structure for phylogenomic inference from supermatrices. Syst Biol. 2016;65:997–1008.

40. Le SQ, Dang CC, Gascuel O. Modeling protein evolution with several amino acid replacement matrices depending on site rates. Mol Biol Evol. 2012;29:2921–36.

41. Hurvich CM, Tsai C-L. Regression and time series model selection in small samples. Biometrika. 1989;76:297–307.

42. Stamatakis A. RAxML version 8: a tool for phylogenetic analysis and post-analysis of large phylogenies. Bioinformatics. 2014;30:1312–3.

43. Guindon S, Dufayard J-F, Lefort V, Anisimova M, Hordijk W, Gascuel O. New algorithms and methods to estimate maximum-likelihood phylogenies: Assessing the performance of PhyML 3.0. Syst Biol. 2010;59:307–21.

44. Anisimova M, Gil M, Dufayard J-F, Dessimoz C, Gascuel O. Survey of branch support methods demonstrates accuracy, power, and robustness of fast likelihood-based approximation schemes. Syst Biol. 2011;60:685–99.

45. Huson DH, Scornavacca C. Dendroscope 3: An interactive tool for rooted phylogenetic trees and networks. Syst Biol. 2012;61:1061–7.

46. Rambaut A. Molecular evolution, phylogenetics and epidemiology: FigTree v1.4.4. 2020. https://github.com/rambaut/figtree. Accessed 1 Jan 2020.

47. Aberer AJ, Krompass D, Stamatakis A. Pruning rogue taxa improves phylogenetic accuracy: an efficient algorithm and webservice. Syst Biol. 2013;62:162–6.

48. Pease JB, Brown JW, Walker JF, Hinchliff CE, Smith SA. Quartet Sampling distinguishes

lack of support from conflicting support in the green plant tree of life. Am J Bot. 2018;105:385–403.

49. Shimodaira H. An approximately unbiased test of phylogenetic tree selection. Syst Biol. 2002;51:492–508.

50. Kishino H, Miyata T, Hasegawa M. Maximum likelihood inference of protein phylogeny and the origin of chloroplasts. J Mol Evol. 1990;31:151–60.

51. Maddison WP, Maddison DR. Mesquite: a modular system for evolutionary analysis. 2019. http://mesquiteproject.org.

52. Strimmer K, von Haeseler A. Likelihood-mapping: a simple method to visualize phylogenetic content of a sequence alignment. Proc Natl Acad Sci U S A. 1997;94:6815–9.

53. Peters RS, Krogmann L, Mayer C, Donath A, Gunkel S, Meusemann K, et al. Evolutionary history of the Hymenoptera. Curr Biol. 2017;27:1013–8.

54. Simon S, Blanke A, Meusemann K. Reanalyzing the Palaeoptera problem – The origin of insect flight remains obscure. Arthropod Struct Dev. 2018;47:328–38.
