# Supplementary material for: Transcriptomics provides a robust framework for the relationships of the major clades of cladobranch sea slugs (Mollusca, Gastropoda, Heterobranchia), but fails to resolve the position of the enigmatic genus Embletonia
Source: BMC Ecol Evol. 2021 Dec 28;21:226. doi: 10.1186/s12862-021-01944-0 (PMC8895541; doi:10.1186/s12862-021-01944-0)
Supplement: Supplementary file 3 — Additional file 3. Figure S1. Species-pairwise site-coverage of the original unreduced and reduced data sets. Heat maps indicate species-pairwise amino acid site-coverage of the sequences of 58 species in the original data sets inferred with AliStat. Low shared site-coverage is in shades of red and high shared site-coverage is in shades of green. AliStat scores are given in Additional file 2: Table S11. a) original unreduced data set. b) original reduced data set. Figure S2. Heat maps calculated with SymTest applying the Bowker’s test on the original unreduced and reduced data sets. Heat maps show the results of pairwise Bowker’s test as implemented in SymTest 2.0.47 analysing the original data sets unreduced and reduced. The percentage of pairwise p-values < 0.05 rejecting SRH conditions are given in parentheses. a) original unreduced data set (p-values < 0.05: 83.36%). b) original reduced data set (p-values < 0.05: 42.65%). Note that especially Calmella and Doris are obvious with respect to violating SRH conditions. Figure S3. Heat map visualising the information content of the final unreduced data set calculated with MARE. The information content (IC) is colour-coded in shades of blue, with darker shades representing higher IC and white squares indicating missing data. Red squares indicate gene partitions with an IC = 0. Species are displayed in rows (x-axis) and gene partitions are displayed in columns (y-axis). Supermatrix diagnostics of MARE are provided in Additional file 2: Table S11. Figure S4. Heat map visualising the information content of the final intermediate data set calculated with MARE. The information content (IC) is colour-coded in shades of blue, with darker shades representing higher IC and white squares indicating missing data. Red squares indicate gene partitions with an IC = 0. Species are displayed in rows (x-axis) and gene partitions are displayed in columns (y-axis). Supermatrix diagnostics of MARE are provided in Additional file 2: Table [file 12862_2021_1944_MOESM3_ESM.pdf]

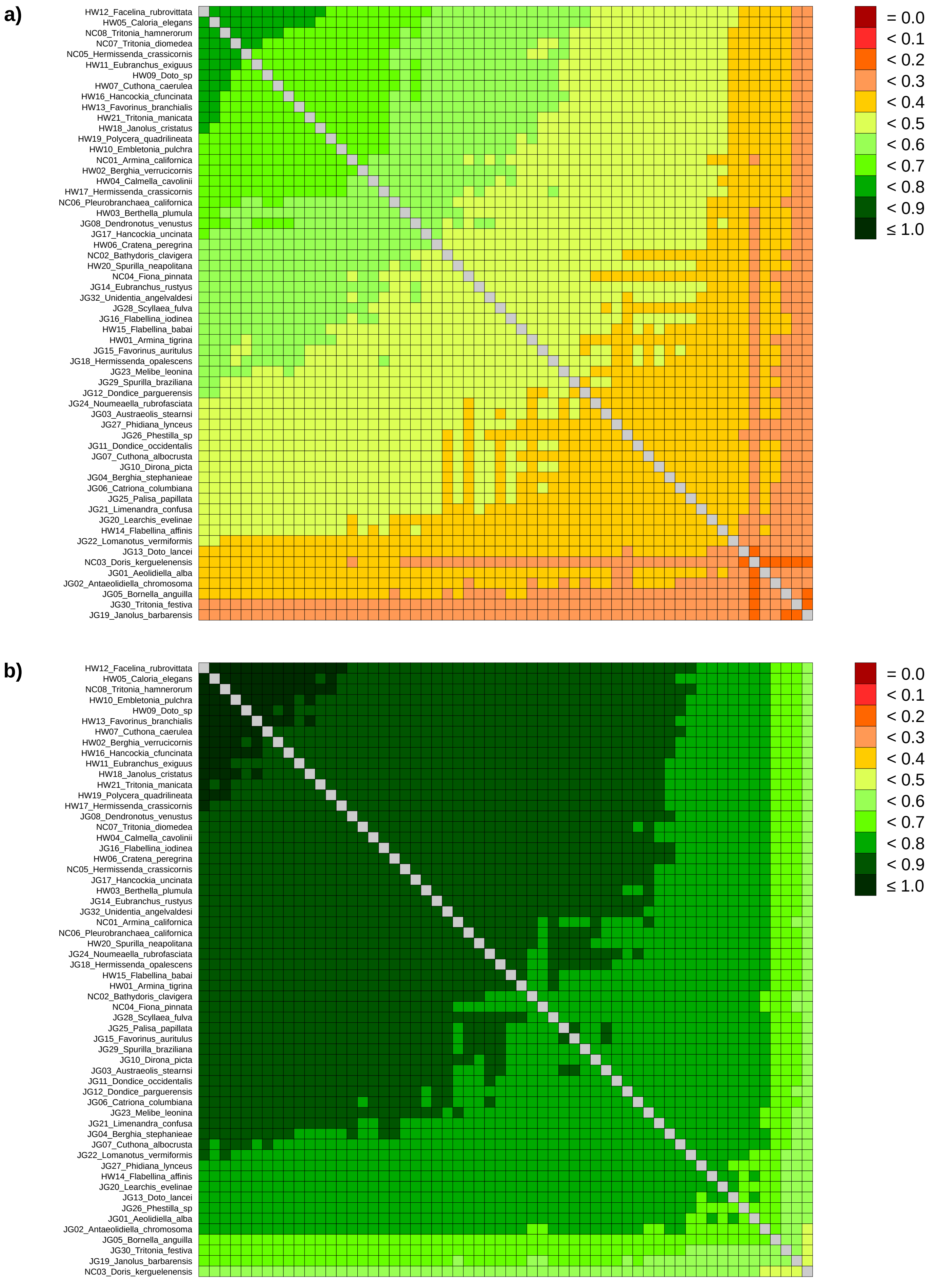


**Figure S1: Species-pairwise site-coverage of the original unreduced and reduced data sets.**
Heat maps indicate species-pairwise amino acid site-coverage of the sequences of 58 species in the original data sets inferred with AliStat. Low shared site-coverage is in shades of red and high shared site-coverage is in shades of green. AliStat scores are given in Supplementary Table S11, Additional File 2. **a)** original unreduced data set. **b)** original reduced data set.

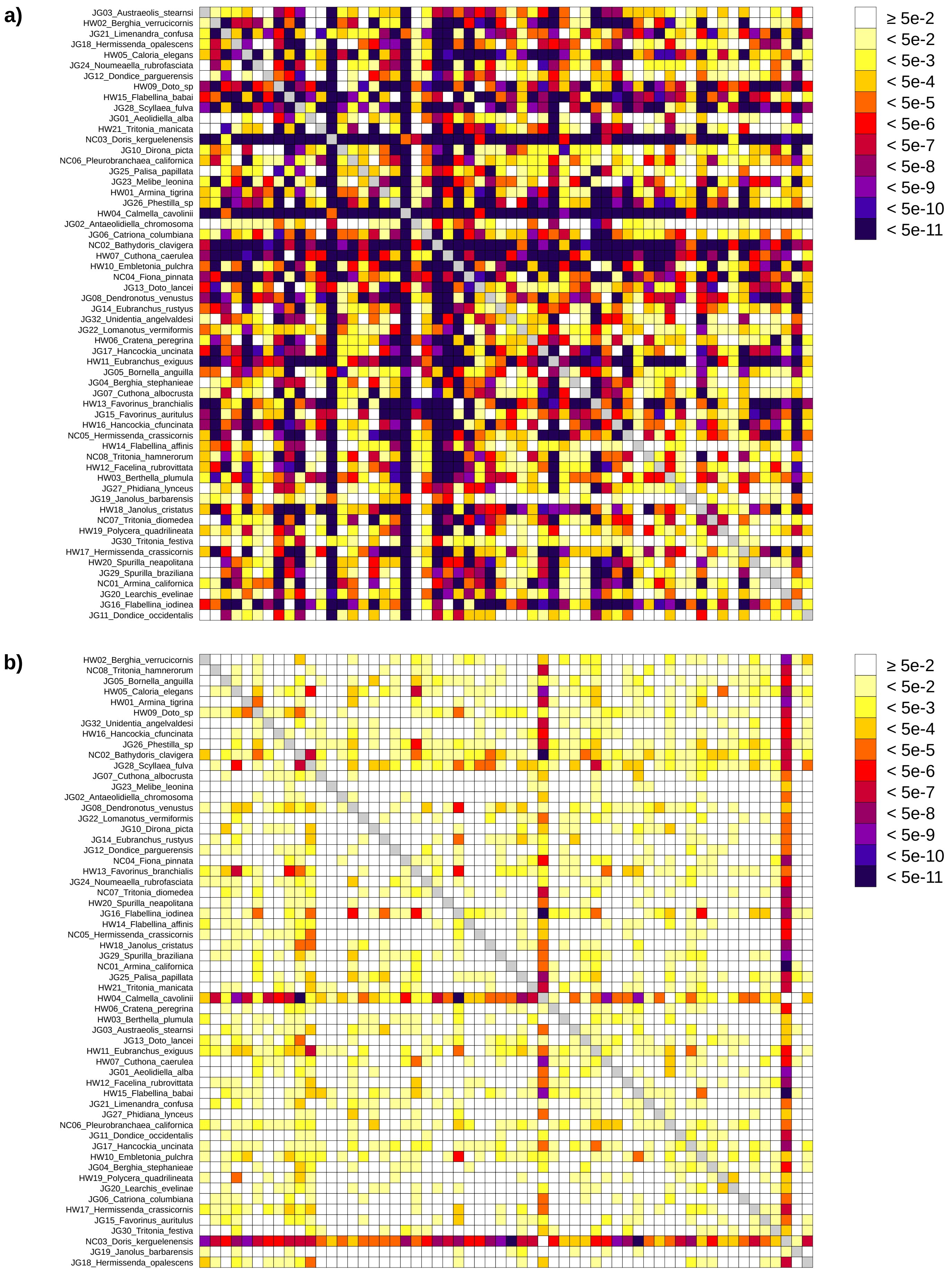


**Figure S2: Heat maps calculated with SymTest applying the Bowker's test on the original unreduced and reduced data sets.**
Heat maps show the results of pairwise Bowker's test as implemented in SymTest 2.0.47 analysing the original data sets unreduced and reduced. The percentage of pairwise p-values < 0.05 rejecting SRH conditions are given in parentheses.
**a)** original unreduced data set (p-values < 0.05: 83.36%). **b)** original reduced data set (p-values < 0.05: 42.65%). Note that especially *Calmella* and *Doris* are obvious with respect to violating SRH conditions.

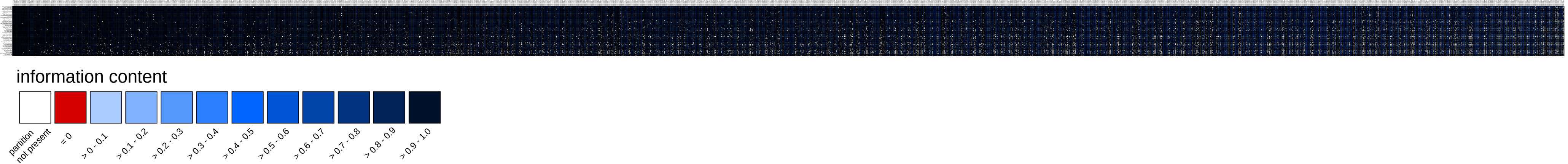


**Figure S3: Heat map visualising the information content of the final unreduced data set calculated with MARE.**
The information content (IC) is colour-coded in shades of blue, with darker shades representing higher IC and white squares indicating missing data. Red squares indicate gene partitions with an IC = 0. Species are displayed in rows (x-axis) and gene partitions are displayed in columns (y-axis). Supermatrix diagnostics of MARE are provided in Supplementary Table S11, Additional File 2.

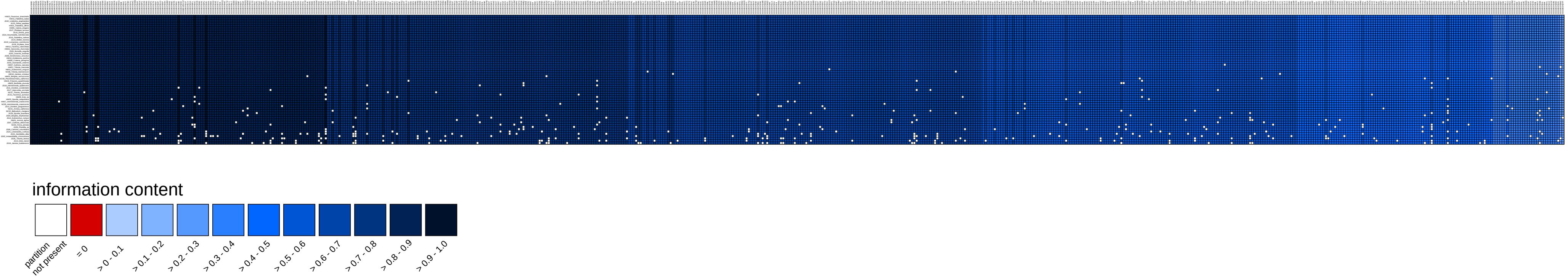


**Figure S4: Heat map visualising the information content of the final intermediate data set calculated with MARE.**
The information content (IC) is colour-coded in shades of blue, with darker shades representing higher IC and white squares indicating missing data. Red squares indicate gene partitions with an IC = 0. Species are displayed in rows (x-axis) and gene partitions are displayed in columns (y-axis). Supermatrix diagnostics of MARE are provided in Supplementary Table S11, Additional File 2.

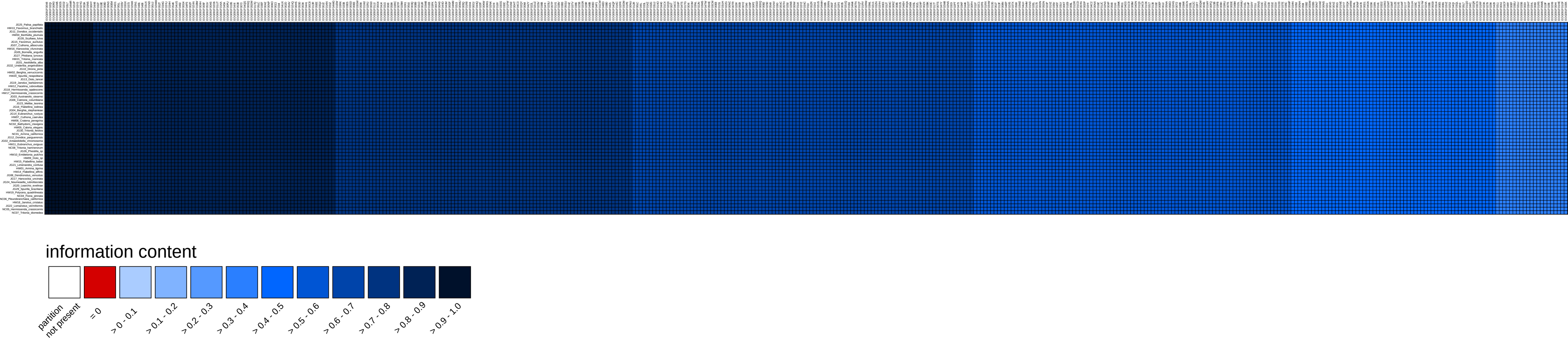


**Figure S5: Heat map visualising the information content of the final strict data set calculated with MARE.**
The information content (IC) is colour-coded in shades of blue, with darker shades representing higher IC and white squares indicating missing data. Red squares indicate gene partitions with an IC = 0. Species are displayed in rows (x-axis) and gene partitions are displayed in columns (y-axis). Supermatrix diagnostics of MARE are provided in Supplementary Table S11, Additional File 2.

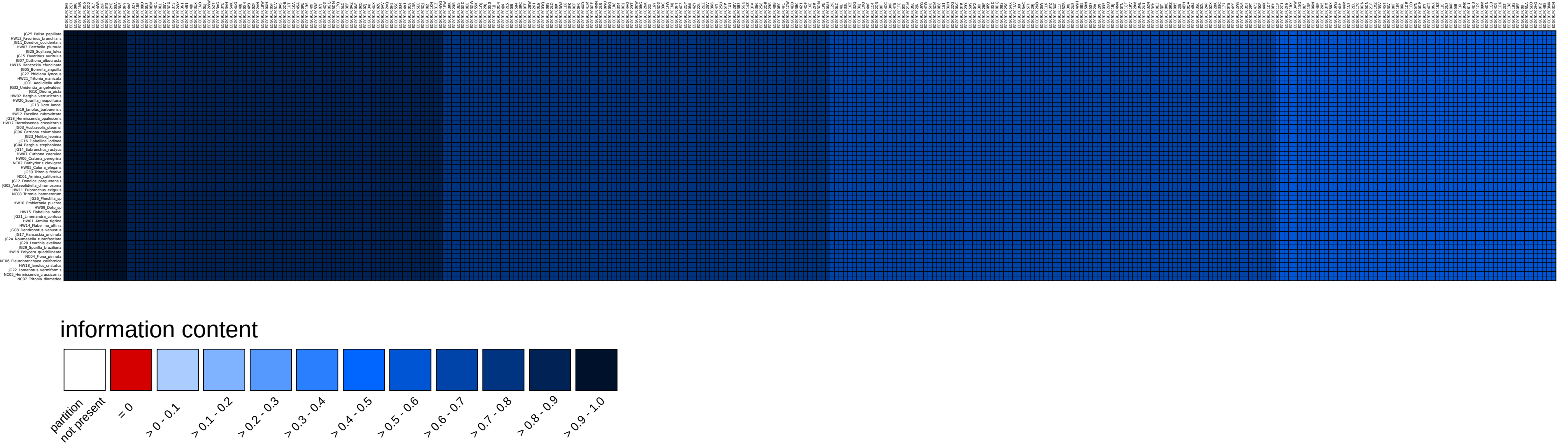


**Figure S6: Heat map visualising the information content of the strict SOS data set calculated with MARE.**
The information content (IC) is colour-coded in shades of blue, with darker shades representing higher IC and white squares indicating missing data. Red squares indicate gene partitions with an IC = 0. Species are displayed in rows (x-axis) and gene partitions are displayed in columns (y-axis). Supermatrix diagnostics of MARE are provided in Supplementary Table S11, Additional File 2.

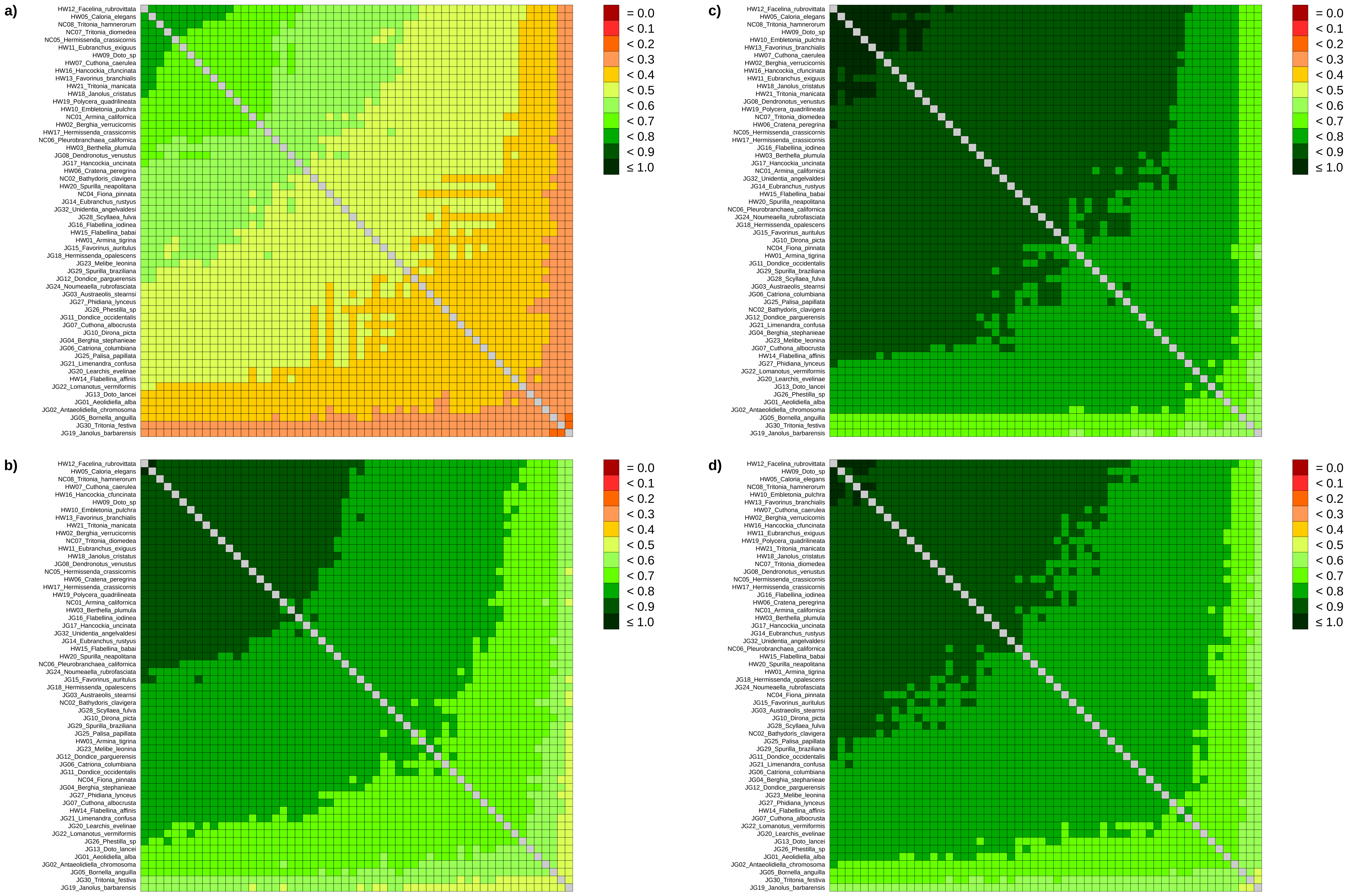


**Figure S7: Species-pairwise site-coverage of the final unreduced, intermediate, strict, and strict SOS data set.**
Heat maps indicate species-pairwise amino acid site-coverage of the sequences of 56 species in the final data sets inferred with AliStat. Low shared site-coverage is in shades of red and high shared site-coverage is in shades of green. AliStat scores are given in Supplementary Table S11, Additional File 2. **a)** unreduced data set. **b)** intermediate data set. **c)** strict data set. **d)** strict SOS data set.

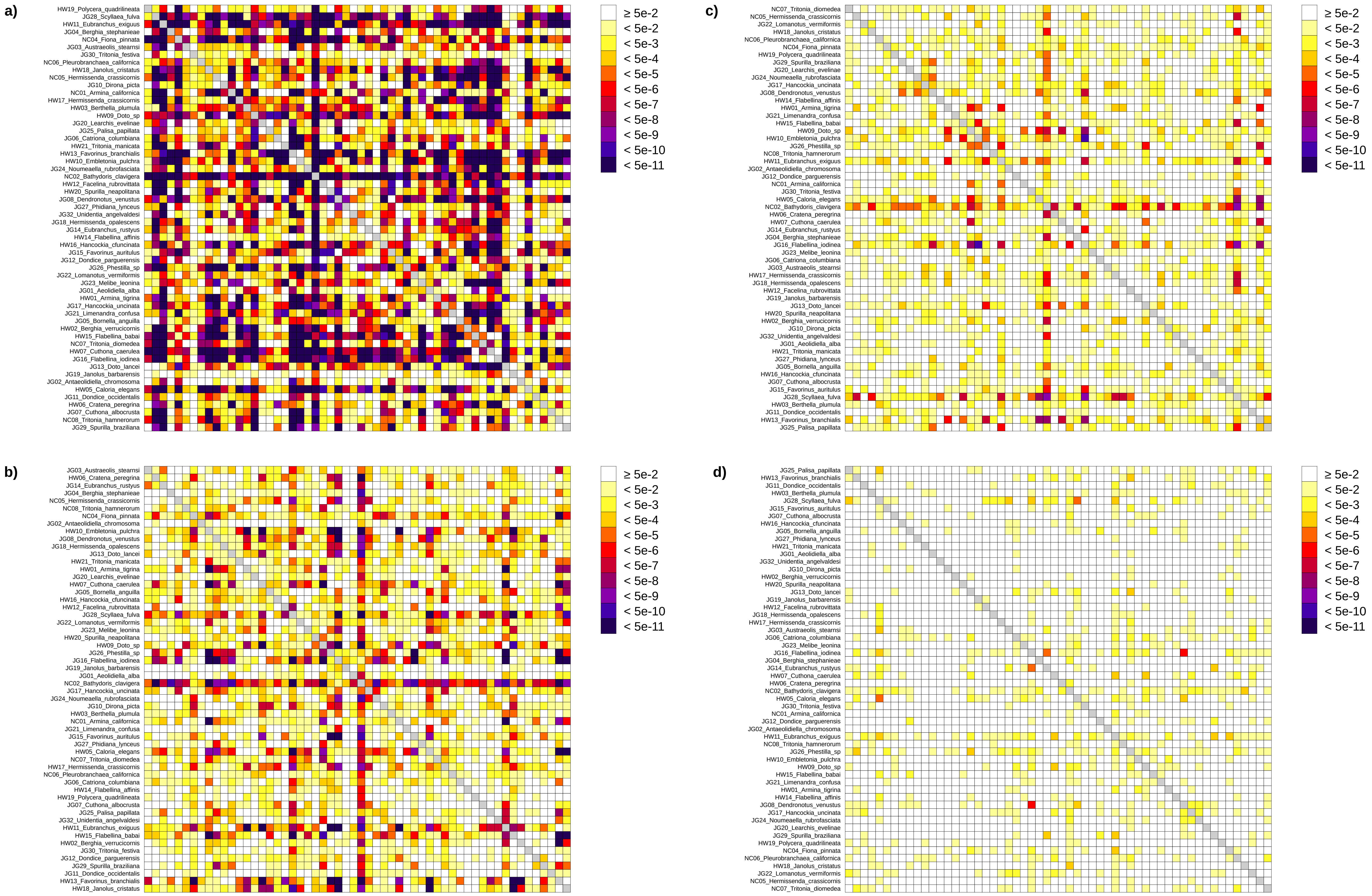


**Figure S8: Heat maps calculated with SymTest applying the Bowker's test on the final unreduced, intermediate, strict, and strict SOS data sets.**
Heat maps show the results of pairwise Bowker's test as implemented in SymTest 2.0.47 analysing the final data sets unreduced, intermediate, strict, and strict SOS. The percentage of pairwise p-values < 0.05 rejecting SRH conditions are given in parentheses. **a)** unreduced data set (p-values < 0.05: 82.14%). **b)** intermediate data set (p-values < 0.05: 63.96%). **c)** strict data set (p-values < 0.05: 46.17%). **d)** strict SOS data set (p-values < 0.05: 21.17%).

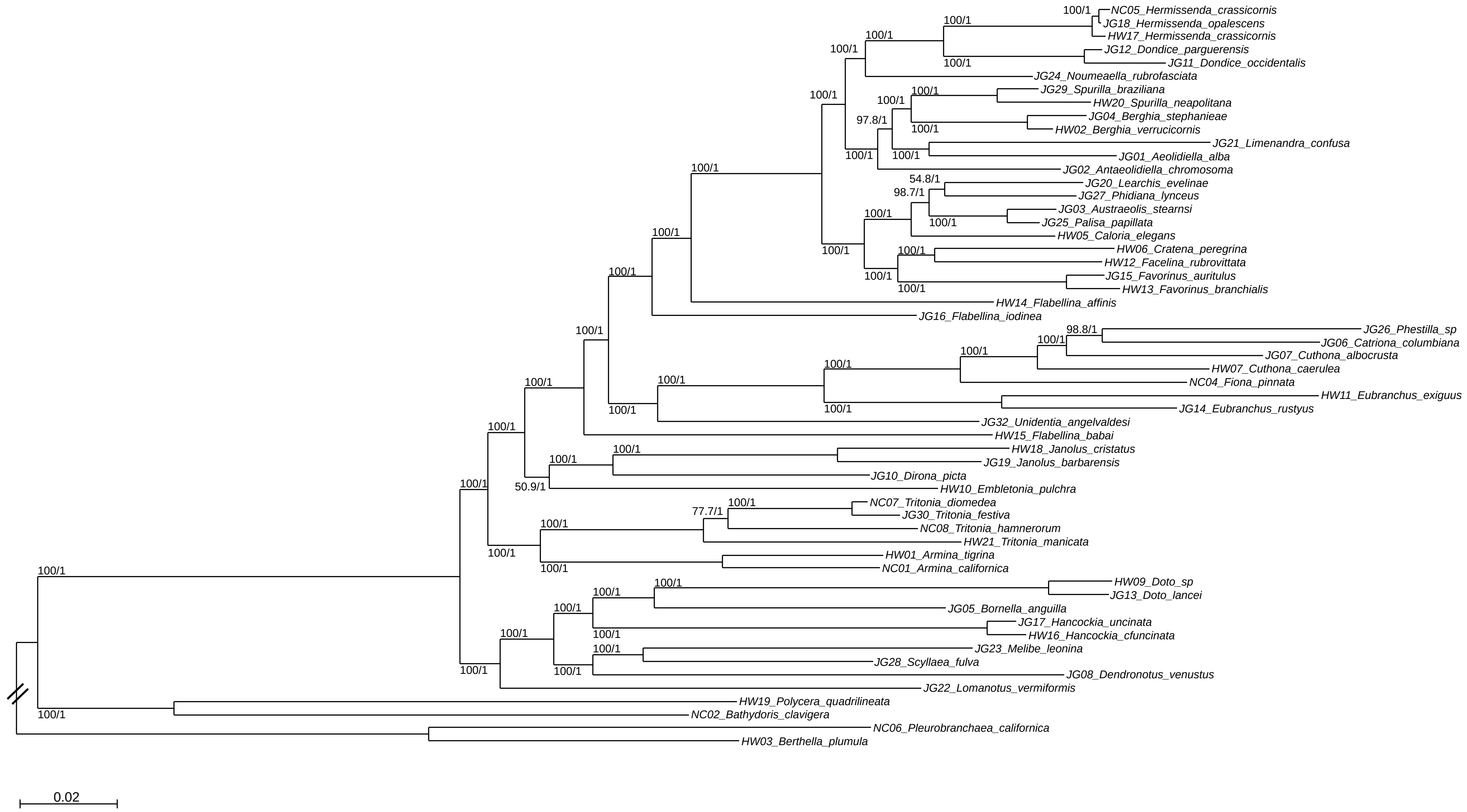


**Figure S9: Best ML tree of the strict data set with aLRT and aBayes support.**
The phylogram is identical to the phylogram in Fig. 1 without the alternative position of Embletonia pulchra. The first value displays branch support based on 10,000 SH-aLRT replicates, the second value displays support derived from the approximate Bayesian support.

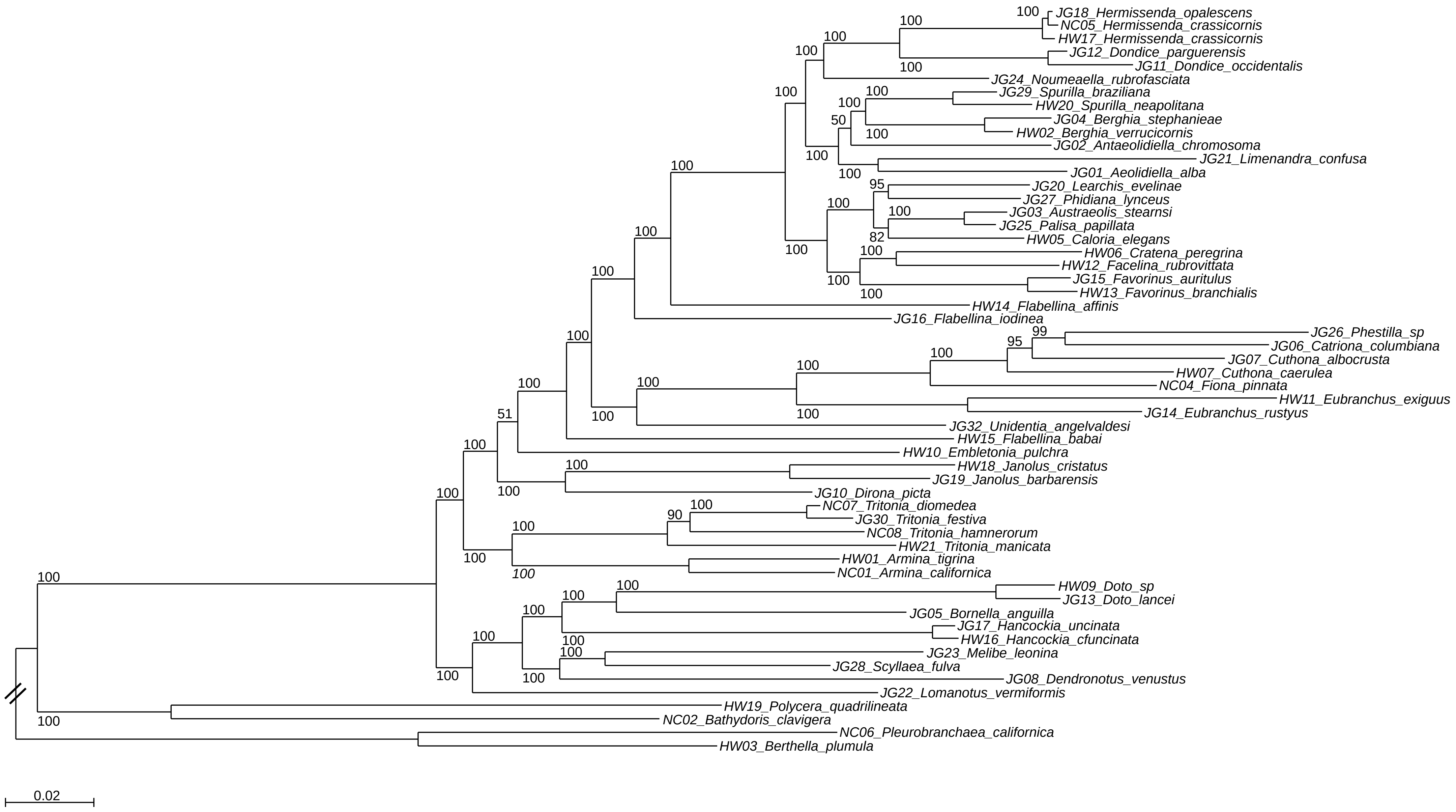


**Figure S10: Best ML tree of the intermediate data set with non-parametric bootstrap support.**
Statistical support was inferred from 300 non-parametric bootstrap replicates.

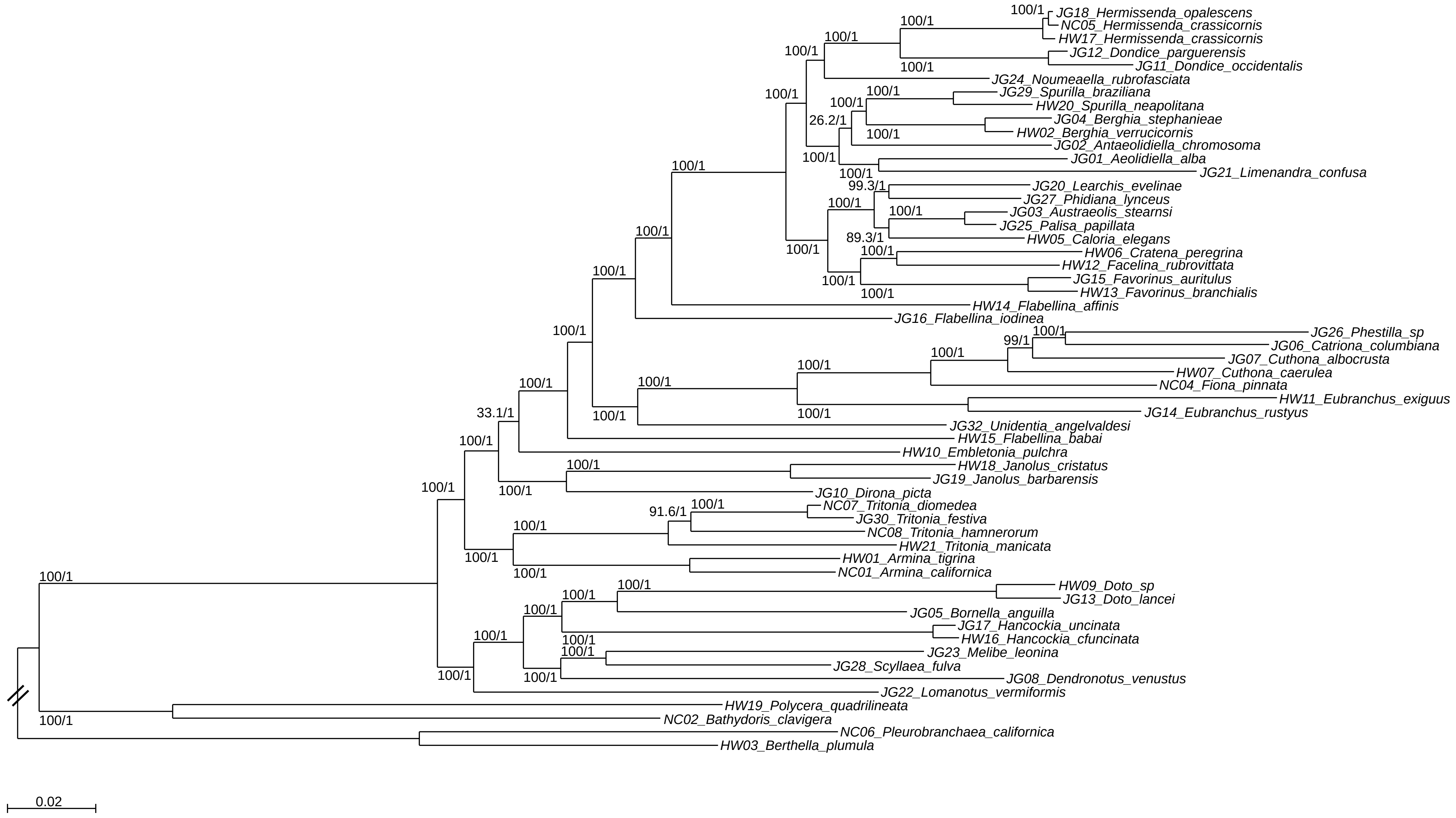


**Figure S11: Best ML tree of the intermediate data set with aLRT and aBayes support.**

The first value displays branch support based on 10,000 SH-aLRT replicates, the second value displays support derived from the approximate Bayesian support.

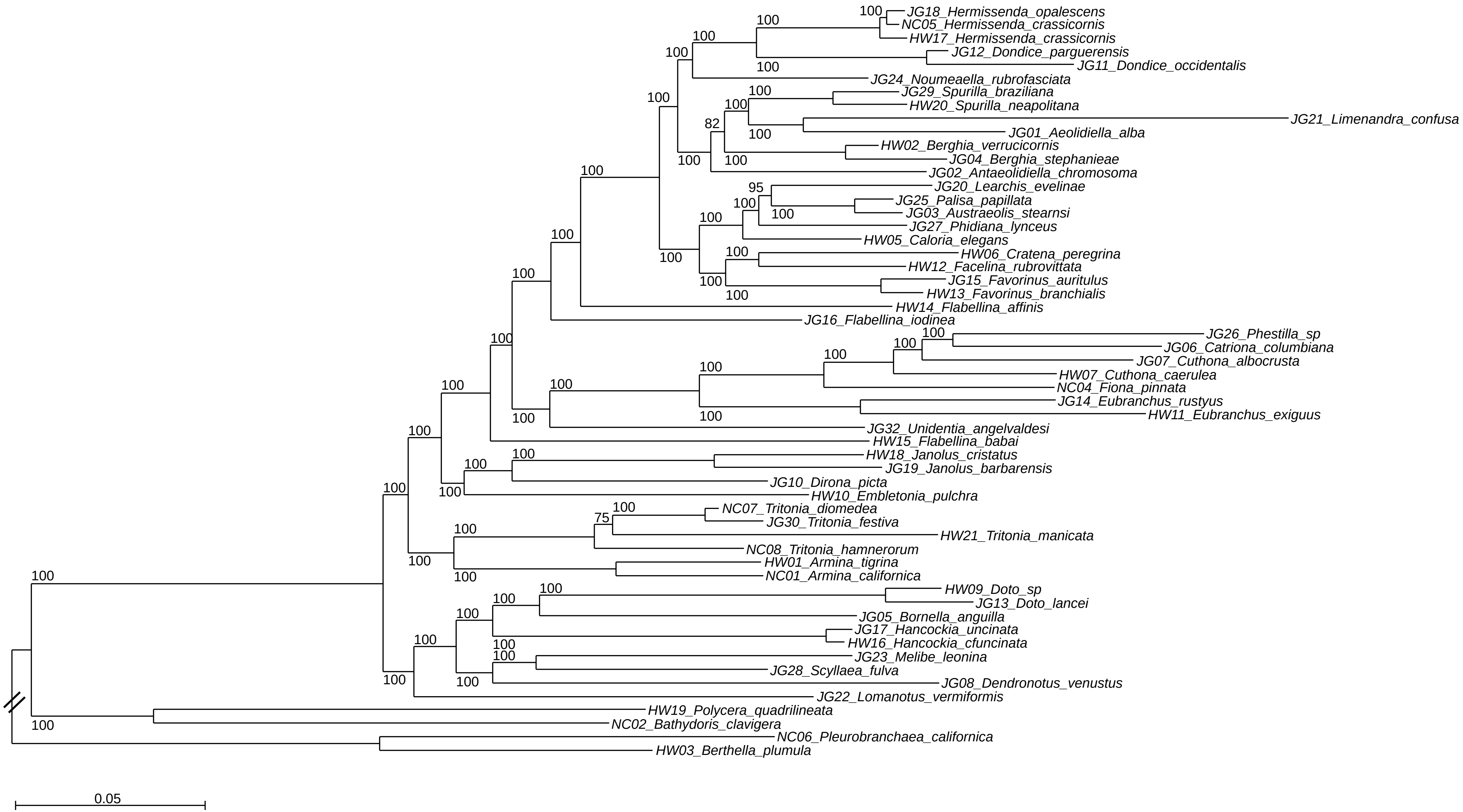


**Figure S12: Best ML tree of the unreduced data set with non-parametric bootstrap support.**
Statistical support was inferred from 100 non-parametric bootstrap replicates.

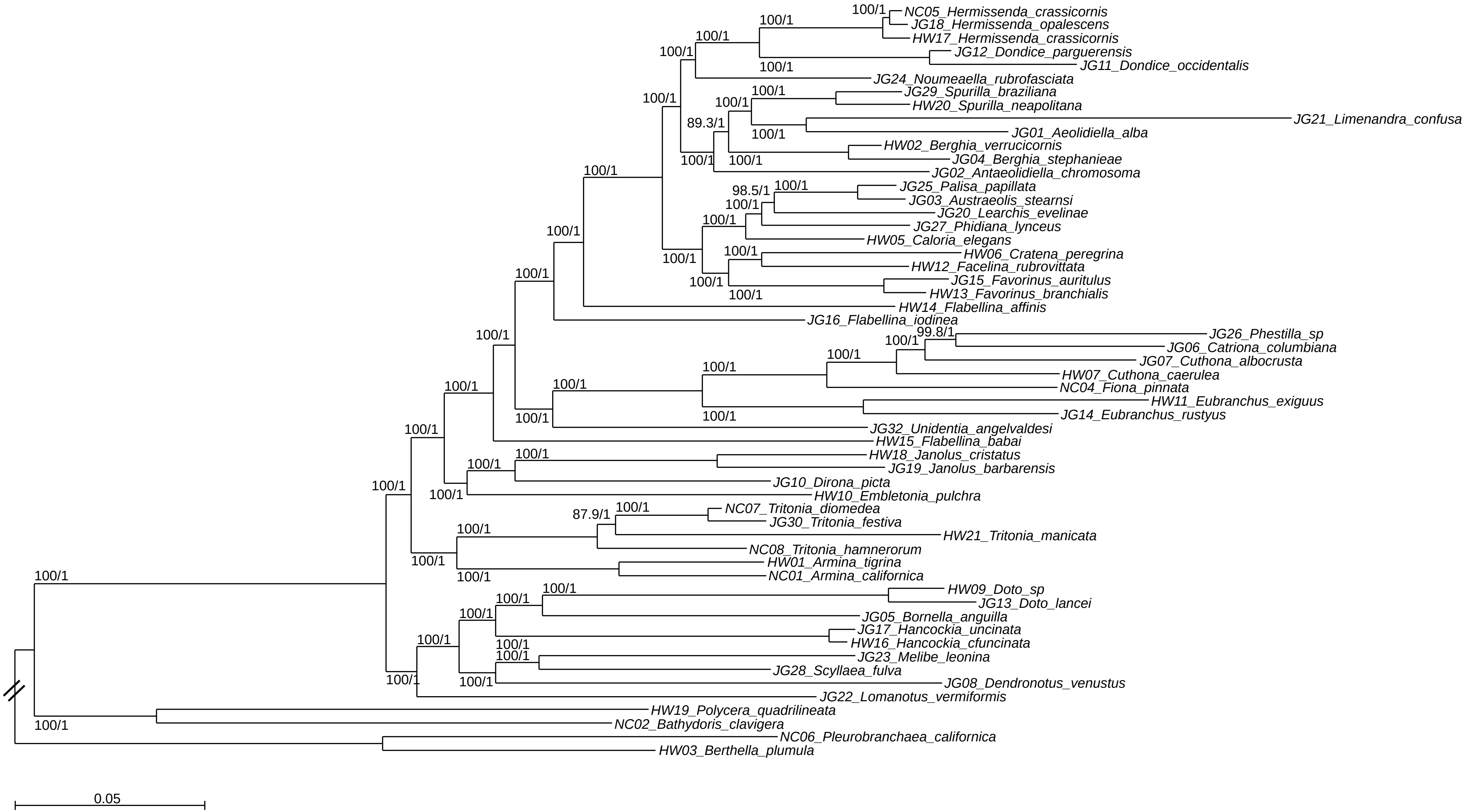


**Figure S13: Best ML tree of the unreduced data set with aLRT and aBayes support.**
The first value displays branch support based on 10,000 SH-aLRT replicates, the second value displays support derived from the approximate Bayesian support.

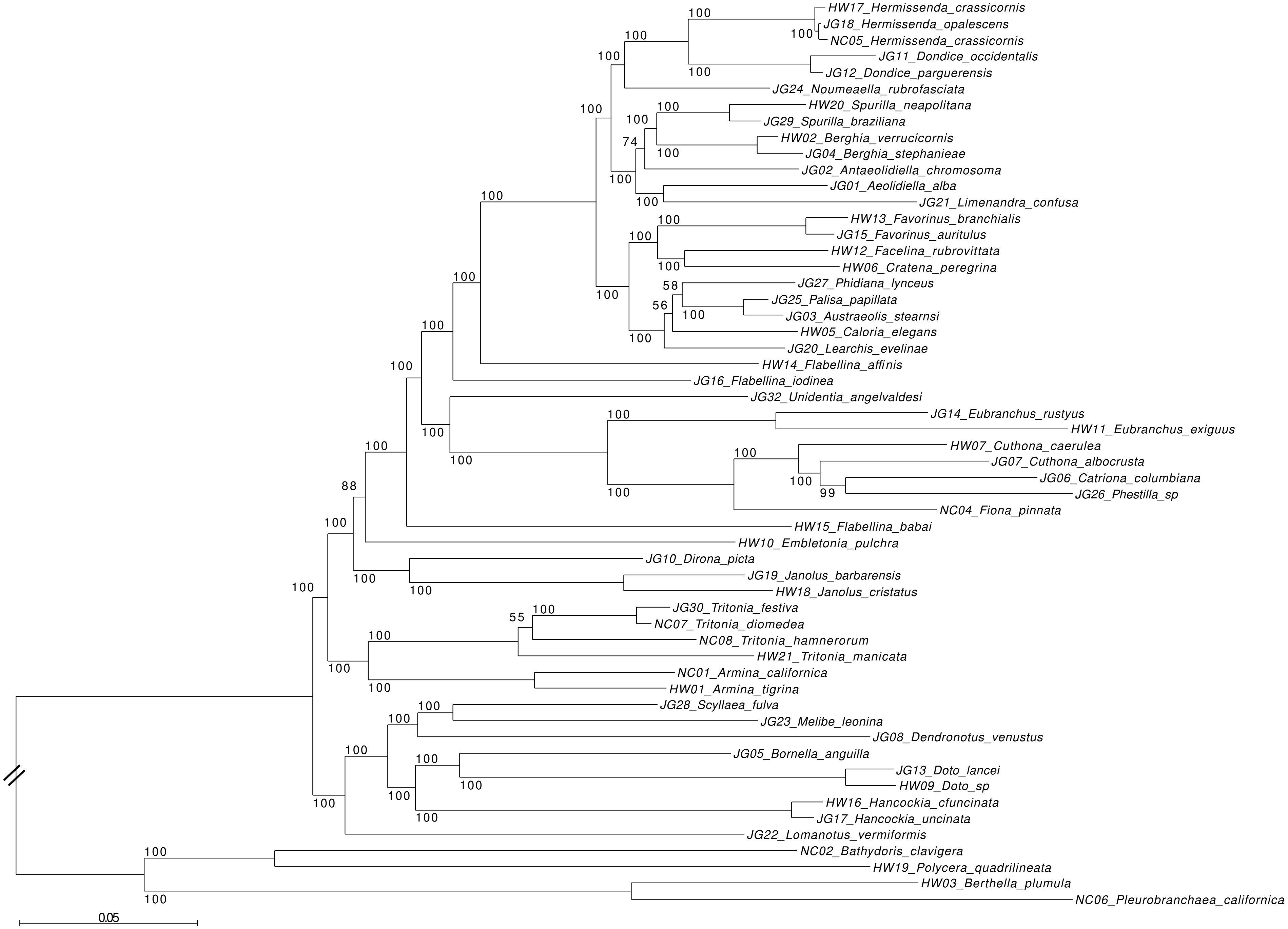


**Figure S14: Best ML tree of the strict unpartitioned data set analysed with a mixture model approach with non-parametric bootstrap support.** Statistical support was inferred from 100 non-parametric bootstrap replicates.

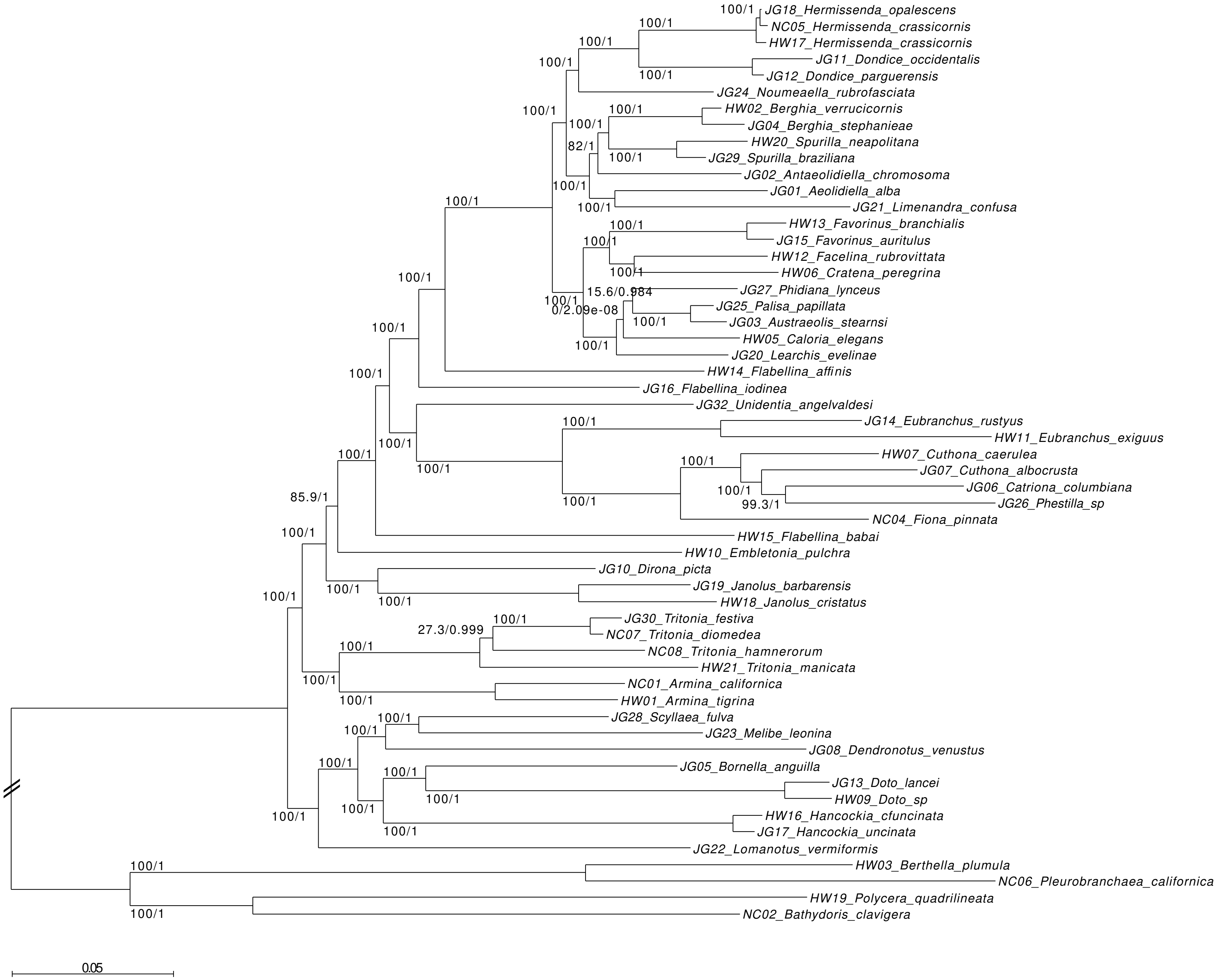


**Figure S15: Best ML tree of the strict unpartitioned data set analysed with a mixture model approach with aLRT and aBayes support.**
The first value displays branch support based on 10,000 SH-aLRT replicates, the second value displays support derived from the approximate Bayesian support.

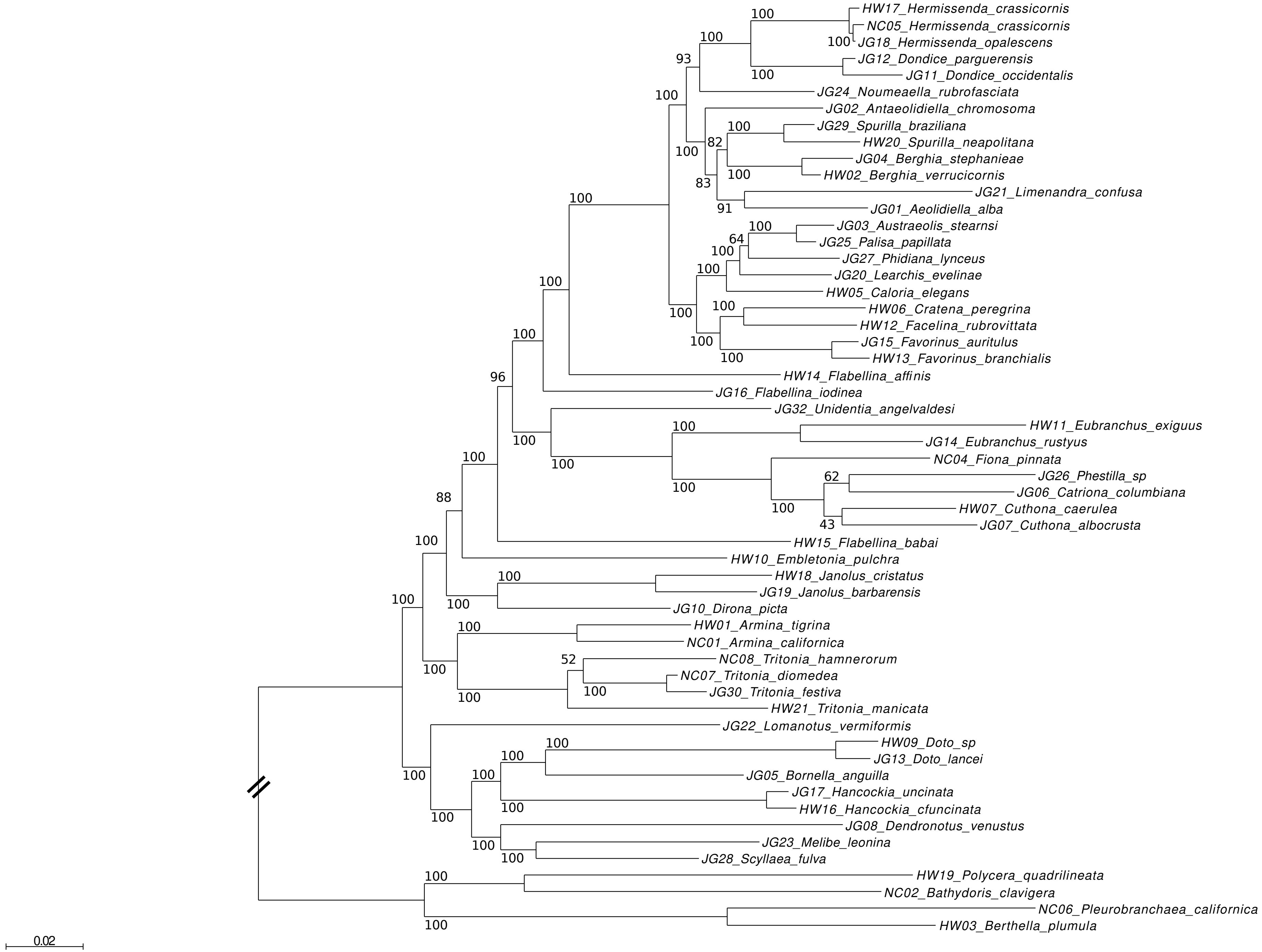


**Figure S16: Best ML tree of the strict SOS data set with non-parametric bootstrap support.**
Statistical support was inferred from 100 non-parametric bootstrap replicates.

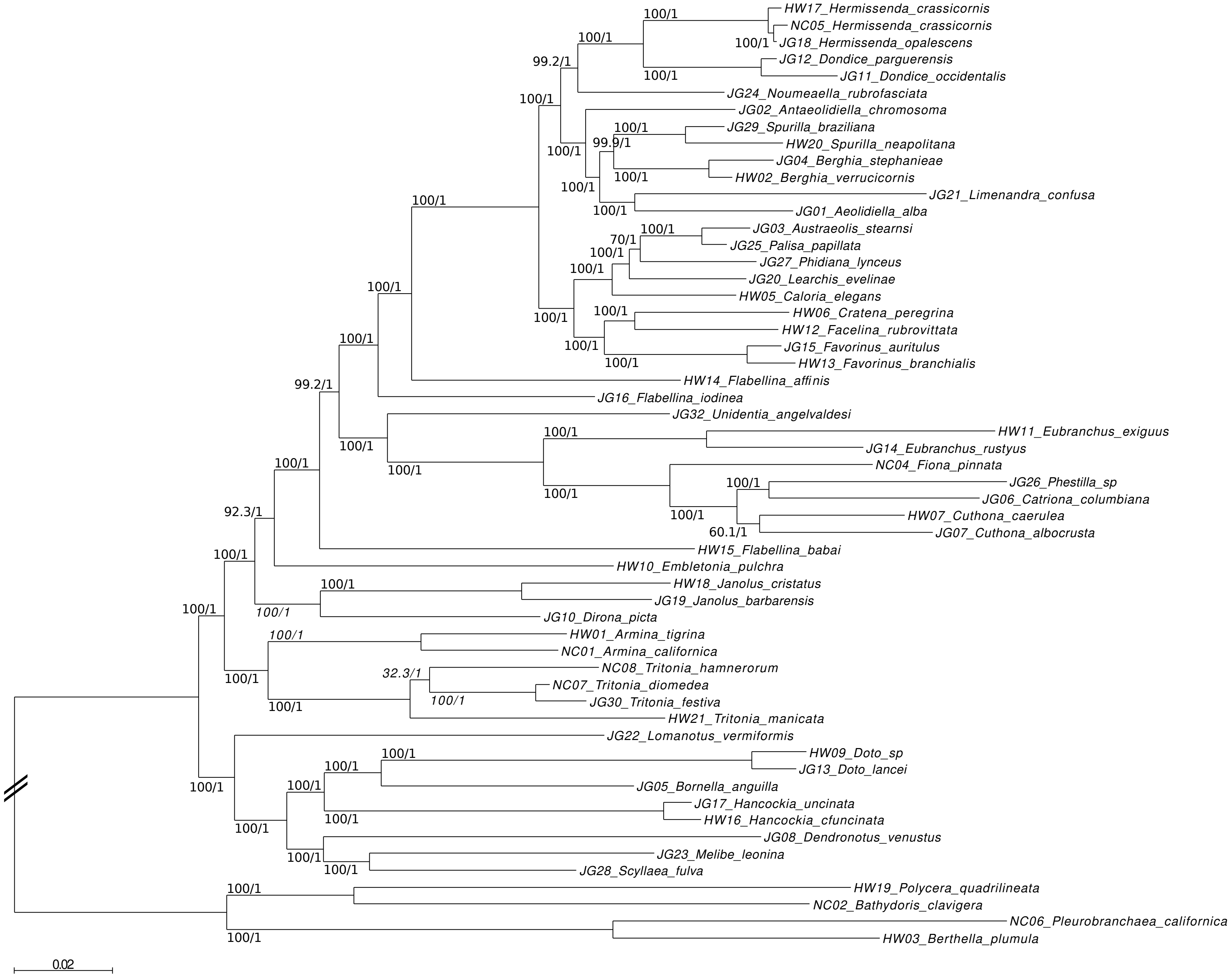


**Figure S17: Best ML tree of the strict SOS data set with aLRT and aBayes support.**
The first value displays branch support based on 10,000 SH-aLRT replicates, the second value displays support derived from the approximate Bayesian support.
